# Draft Genome Sequence of Enterobacter cloacae 3F11 (Phylum *Proteobacteria*)

**DOI:** 10.1128/MRA.00846-18

**Published:** 2018-08-16

**Authors:** Christopher R. Dumigan, Gregory E. Perry, K. Peter Pauls, Manish N. Raizada

**Affiliations:** a Department of Plant Agriculture, University of Guelph, Guelph, Ontario, Canada; Loyola University Chicago

## Abstract

Presented here is the draft genome sequence of Enterobacter cloacae 3F11. This seed endophyte solubilizes rock phosphate and was isolated from Zea nicaraguensis. The genome contains 4,579,108 bp in 264 contigs.

## Abstract (precis)

Presented here is the draft genome sequence of Enterobacter cloacae 3F11. This seed endophyte solubilizes rock phosphate and was isolated from Zea nicaraguensis.

## ANNOUNCEMENT

The insolubility of rock phosphate presents a major challenge for plants to obtain this vital macronutrient (1). Certain forms of rock phosphate can be made bioavailable by acidification, and some plant species have evolved organic acid excretion as an adaptation to soils rich in rock phosphate (2). Plants can also adapt to their environment by hosting nonpathogenic microbes known as endophytes that provide a selective advantage to their host (3).

A recent study identified an endophytic Enterobacter sp. in Zea nicaraguensis, a wild maize growing in a swamp at the base of the San Cristobal Volcano in Nicaragua (4). The primary form of phosphate in volcanic rock is highly insoluble. This endophytic Enterobacter species was found to localize to the root hair cells of modern corn and annual ryegrass (a model grass relative of corn), increase the root growth of annual ryegrass on insoluble rock phosphate, and secrete acids to solubilize rock phosphate (4).

Here, we present the whole-genome sequence of Enterobacter sp. strain 3F11, which was isolated from surface-sterilized seeds of Zea nicaraguensis in 2011 (5). Genomic DNA was isolated from a 30°C (200 rpm) overnight lysogeny broth (LB) culture grown from a single colony using a Norgen bacterial genomic DNA isolation kit (catalog number 17900; Thorold). A paired-end library was created using a Nextera XT DNA library prep kit (Illumina, San Diego, CA), and sequencing was performed on an Illumina MiSeq platform. The analysis generated 1,298,802 read pairs averaging 151 bp in size. The total genome coverage was 43-fold, and 1,297,520 reads remained after trimming low-quality reads. *De novo* assembly was performed using CLC Genomics Workbench 10.0.1 (Qiagen) and yielded a genome size of 4,579,108 bp in 264 contigs (minimum, 1,059 bp; minimum, 124,272 bp; *N*_50_, 29,224 bp), with a GC content of 56.4%.

Genome annotation, carried out by the RAST server (6), identified 4,201 protein-encoding genes, 58 tRNA operons, and 3 rRNA operons. Taxonomy was determined using K-mer finder (7, 8), and the closest match was found to be Enterobacter cloacae, with 98.6% nucleotide similarity to Hoffman complex IV (9); thus, we propose the strain name E. cloacae IV 3F11.

### Data availability.

The results of the whole-genome shotgun project were deposited at DDBJ/EMBL/GenBank under the accession number QKVR00000000. The version described in this paper is version QKVR01000000. Raw Illumina reads are available at SRA accession number SRR7310671.
